# Assessment of the P Wave Dispersion and Duration in Elite Women Basketball Players

**Published:** 2010-01-07

**Authors:** Gokhan Metin, Mustafa Yildiz, Bulent Bayraktar, Ilker Yucesir, Hasan Kasap, Lutfi Cakar

**Affiliations:** 1 Department of Physiology, Istanbul University Cerrahpasa Medical Faculty, Istanbul, Turkey; 2 Department of Cardiology, Kartal Kosuyolu Yuksek Ihtisas Educational and Research Hospital, Istanbul, Turkey; 3 Department of Sports Medicine, Istanbul University Medical Faculty, Istanbul, Turkey; 4 School of Physical Education and Sports , Istanbul University, Istanbul, Turkey; 5 Department of Cardiology, Umut Hospital, Istanbul, Turkey

**Keywords:** P wave dispersion, echocardiography, left atrium, atrial fibrillation, athletes

## Abstract

**Background:**

P wave dispersion is an independent predictor of atrial fibrillation. P wave dispersion is associated with inhomogeneous and discontinuous propagation of sinus impulses. The purpose of this study was to investigate P wave dispersion and transthoracic echocardiographic findings in elite women basketball players.

**Methods:**

We recruited 27 well-trained woman athletes with a training history of many years (11.9 ± 3.6 years). All of the athletes were elite women basketball players and they were regularly maintaining sportive activities and training programs. Twenty-six age and sex matched healthy sedentary subjects consisted of control group. The difference between P maximum and P minimum durations was defined as P wave dispersion. The echocardiographic parameters were assessed in detail in the standard left lateral decubitus position.

**Results:**

The body height, body weight, body surface area, metabolic equivalent, maximum P wave duration and P wave dispersion were increased in the elite basketball athletes as compared with healthy sedentary subjects. On the contrary; the heart rate, ejection fraction and interventricular septum thickness in diastole were decreased in athletes. The body height (p=0.006, r=0.37), body weight (p=0.04, r=0.28), body surface area (p=0.01, r=0.33) and heart rate (p=0.01, r=-0.32) were correlated with P wave dispersion.

**Conclusions:**

P wave dispersion was increased in elite woman basketball players as compared with healthy sedentary subjects. P wave dispersion was correlated with heart rate, body height, body weight and body surface area.

##  Introduction

Basketball is one of the most popular and widely viewed sports in the world. It is a team sport in which two teams of five active players each try to score points against one another by placing a ball through a 10 feet high hoop under organized rules. In this sport, players cover about 4500–5000 m during a 40-min game with a variety of multidirectional movements such as running, shuffling and dribbling at variable velocities and jumping [1]. To execute such movements during performance, both aerobic and anaerobic metabolic systems appear to be involved throughout a game [2].

Widely used in sports medicine, the transthoracic echocardiography allows quantitative assessment of cardiac structure and function for the athletes. Changes in cardiac parameters due to dynamic exercise in normal subjects have been well documented by many published echocardiographic studies [3,4]. These articles show that well trained athletes had greater chamber dilation and increased left ventricular mass compared with nonathletes. It was thought that such changes were secondary to physiologic adaptation from heavy exercise. As specific features of the basketball player's myocardial structure and function are still rather poorly investigated. Vasiliauskas et al [5] demonstrated that regular basketball training results in moderate cardiac hypertrophy in adolescents and adult athletes due to thickening of myocardial walls. Wolfe et al [6] showed that left ventricular internal dimension, posterior wall thickness, septal thickness, and calculated left ventricular mass in the elite college basketball players were within or only slightly in excess of echocardiographic normal limits and mean values were not significantly different from the control group. Left ventricular internal dimension (mm/m^2^ body surface area) was significantly lower in the elite college basketball players. However, five guard-type players displayed significantly greater mean values for posterior wall thickness and left ventricular mass compared to six taller forwards/centres with linear body builds. Karagoz et al [7] showed that an upper extremity exercise program and sports such as basketball can improve the cardiac functions and additional echocardiographic functions such as left ventricular ejection fraction and shortening fraction of people unable to use their lower extremities, potentially to normal levels.

The elite athletes often exhibit some changes in the heart, called athlete's heart [8]. Athlete's heart is an enlarged heart related to repeated strenuous exercise. As a result of exercise, the heart will expand physiologically by enlarging chambers, increasing muscle mass and increasing the volume of blood pumped per stroke. The athlete's heart is associated with some types of electrocardiogram (ECG) abnormalities, such as sinus bradycardia, sinus arrhythmia, early repolarization, first-degree atrioventricular block and left atrial enlargement [9].

Atrial fibrillation is the most frequent cause of prolonged palpitations in young competitive athletes, even including those performing elite sport activity [10]. Endurance sport practice increases the probability of suffering atrial fibrillation, after adjusting for other risk factors [11]. The possible mechanisms explaining the association remain speculative. Atrial ectopic beats, inflammatory changes, and atrial size have been suggested [11]. In patients with hypertension or structural heart disease, it seems that atrial fibrillation is the consequence of structural changes in the atria (dilatation and fibrosis) secondary to chronic volume and pressure overload. It is therefore plausible that long-term endurance sport practice or occupational physical activity may induce structural changes in the atrium (enlargement, fibrosis) that may create a favourable substrate for the disease  [11]. A recent study by Swanson [12] shows that excessive endurance exercise and overtraining can lead to chronic systemic inflammation, inflammation may be mediated by the renin-angiotensin system and a sustained increase in catecholamines, and there is a relationship between atrial fibrillation and C-reactive protein. Anti-inflammatory agents may decrease the C-reactive protein and ameliorate atrial fibrillation. Pelliccia et al  [13] found that atrial size was larger in athletes than in controls, and this was a predictor for atrial fibrillation. Other proposed mechanisms are increased vagal tone, bradycardia and gastroesophageal reflux (it has not yet been properly investigated) [14-17]. Competitive sport has a significant impact on the autonomous nervous system. In fact, long-term regular intense physical training determines an increase in vagal tone leading to resting bradycardia. During physical activity, particularly in the setting of competition, a marked release of catecholamines occurs as a result of both the intense physical effort and emotional stress [10]. Both of these adaptive phenomena may precipitate atrial fibrillation. Experimental data show that increased vagal tone shortens and increases the dispersion of the atrial refractory period, creating the conditions for re-entry [16,17].

P wave dispersion, detected from the surface ECG, have been thought to reflect left atrial enlargement and altered conduction [18]. P wave dispersion and P wave maximal duration, reflects the activation of atrial muscle and may depend primarily upon the mass of tissue excited, have been used in the assessment of the risk for atrial fibrillation which is characterized by nonhomogeneous and discontinuous atrial conduction [19,20]. P wave dispersion was defined as the difference between the longest and the shortest P wave duration recorded from multiple surface ECG leads [19]. The clinical significance of P wave duration has been demonstrated in many clinical conditions, especially in paroxysmal atrial fibrillation [19,21]. P wave dispersion has been showed to be influenced by the autonomic nervous system activation, which induces changes in left atrial size and the velocity of impulse propagation [22,23]. No studies are cited in the literature to suggest the women basketball athletes are at increased risk for atrial fibrillation. The purpose of this study was to investigate P wave dispersion and transthoracic echocardiographic findings in elite women basketball players.

## Methods

### Subjects  population

We recruited 27 well-trained woman athletes with a regular training history of a long period [11.9 ± 3.6 years; mean metabolic equivalent (MET): 14.48 ± 0.68 kcal/kg/hr (min 13.60- max 15.00)]. All of athletes were of same ethnicity (Turkish ethnicity) and were elite women basketball players and they were regularly maintaining sportive activities and training programs. Twenty-six age and sex matched healthy sedentary subjects build up control group. A detailed history was taken and each participant underwent a systemic physical examination to exclude cardiovascular or other relevant disease before participating in the study. Any subject, who had a history of cardiovascular or any other systemic disorders such as hyperlipidemia, hyperglycemia, anemia and those on medications known to alter cardiac conduction were excluded from the study. All subjects were non smokers and don't use alcohol. All subjects gave their consent for inclusion in the study. The investigation conforms to the principles outlined in the Declaration of Helsinki. The study was approved by the ethics committee of Cerrahpasa Medical Faculty of the University of Istanbul.

### Body mass index measurement

Body mass index (kg/m^²^) was calculated dividing body weight in kilograms by square of body height in meters.

### Body surface area measurement

Body surface area (m^²^) = 0.007184 x Height (cm)^0.725^ x Weight (kg)^0.425^  [24].

### Blood pressure measurement

The arterial blood pressure of the subjects was measured by the same clinician. The subjects were in supine position and had rested at least 20 minutes before the measurement. The blood pressure was measured, using a mercury sphygmomanometer with a cuff appropriate to the arm circumference (Korotkoff phase I for systolic blood pressure and V for diastolic blood pressure). Blood pressure measurements were performed twice for each subject and their mean was used for statistical analysis.

Pulse pressure = systolic blood pressure - diastolic blood pressure

Mean blood pressure = [systolic blood pressure + 2 X diastolic blood pressure] / 3

### P wave dispersion measurement

P wave duration was measured in all simultaneously recorded 12 leads of the surface ECG. All recordings were performed in the same quiet room during spontaneous breathing, following 20 minute of adjustment in the supine position. P wave duration measurements were obtained manually by two of the investigators using calipers and magnifying lens for accurate definition of the ECG deflection as defined in a previous study [19]. The onset of the P wave was defined as the point of the first visible upward departure of the trace from the bottom of the baseline. The return to the baseline of the bottom of the trace in wave was considered to be the end of the P wave. P maximum in any of the 12 lead surface ECGs was measured and used as a marker of prolonged atrial conduction time. The difference between P wave maximum and P wave minimum durations was defined as P wave dispersion.

### Transthoracic Echocardiography

The left atrial diameter, left ventricle diastolic diameter, left ventricle systolic diameter, ejection fraction, interventricular septum thickness in diastole, left ventricle posterior wall thickness in diastole and valvular structures were assessed in detail using the standard left lateral decubitus position with Vivid 3 cardiovascular ultrasound system [3S sector probe (1.5-3.6 MHz), GE]  [25]. The left atrial diameter, left ventricle diastolic diameter, left ventricle systolic diameter, interventricular septum thickness in diastole and left ventricle posterior wall thickness in diastole were corrected for body surface area.

### Statistical analysis

The statistical analysis was done using the SPSS (version 8.0) ready-to-use programme. All values were expressed as mean ± standard deviation. Mann-Whitney U non-parametric and Pearson correlation tests were done. Significance limit was accepted as p ≤  0.05.

## Results

The height, weight, body surface area, MET, maximum P wave duration and P wave dispersion were increased in the elite basketball athletes as compared with healthy sedentary subjects. On the contrary; the heart rate, ejection fraction and interventricular septum thickness in diastole were decreased in athletes (Table 1). Pearson correlation analysis of P wave dispersions of all subjects with the parameters; age (p=0.37, r=0.12), body mass index (p=0.56, r=-0.08), systolic blood pressure (p=0.54, r=0.08), diastolic blood pressure (p=0.71, r=-0.05), pulse pressure (p=0.23, r=0.16), mean blood pressure (p=0.96, r=-0.006), left atrial diameter (p=0.24, r=-0.16), left ventricle diastolic diameter (p=0.58, r=-0.07), left ventricle systolic diameter (p=0.18, r=-0.18), ejection fraction (p=0.10, r=-0.22), inter-ventricular septum thickness in diastole (p=0.26, r=-0.15) and left ventricle posterior wall thickness in diastole (p=0.53, r=0.08) showed no significant relation. The only relation was found with body height (p=0.006, r=0.37), body weight (p=0.04, r=0.28), body surface area (p=0.01, r=0.33) and heart rate (p=0.01, r=-0.32).

## Discussion

We found that the body height, body weight, body surface area, maximum P wave duration and P wave dispersion were increased in the elite basketball athletes as compared with healthy sedentary subjects. On the contrary; the heart rate and ejection fraction were decreased in the athletes. Although the body height, body weight and body surface area were positively associated with P wave dispersion, the heart rate was negatively associated with P wave dispersion.

Long-term athletic conditioning is associated with cardiac morphologic changes, including increased left ventricle systolic and diastolic diameter, wall thickness and mass [13]. The increase in ejection fraction on dynamic exercise was not different from control in elite athletes [26-29]. However, fractional shortening or ejection fraction at rest was significantly higher or depressed (within normal limits) in athletes [30,31], as we found in our study. High intensity dynamic endurance exercise is usually associated with the rhythm and conduction abnormalities that result from the bradycardia, changes in autonomic nervous system [9]. Therefore, a variety of ECG abnormalities can be found in trained athletes. Recently, Crouse et al [9] demonstrated that abnormal resting ECG findings e.g. left ventricular hypertrophy (64.5%), interventricular conduction delay (2.6%), sinus bradycardia (9.1%), sinus arrhythmia (15.6%), first-degree atrioventricular block (11.7%), early repolarization (33.8%), and left atrial enlargement (48.1%) are common in a sample of collegiate football athletes.

A number of cardiac arrhythmias have been described in athletes [9]. Among these are sinus bradycardia, as we found in our study, and varying degrees of atrioventricular block due to high resting vagal tone [30,31]. It has also been suggested that there are intrinsic changes within the sinoatrial and atrioventricular nodes, including prolonged sinus node recovery time. It is thought that athletes are vulnarable to atrial fibrillation because of enhanced parasympathetic activity and consequent bradycardia is likely to cause dispersion of atrial repolarization [32,33]. This dispersion of atrial repolarization can facilitate atrial fibrillation [34]. P wave dispersion, an electrocardiographic marker, is an independent predictor of atrial fibrillation. P wave dispersion is associated with inhomogeneous and discontinuous propagation of sinus impulses [35]. The nonhomogeneous propagation of sinus impulses and the prolongation of atrial conduction time are electrophysiologic characteristics in patients with atrial fibrillation [35]. Some studies showed that endurance-trained subjects present higher values of power spectral heart rate variability (HRV), indicating increasing vagal activity [35-38]. Chen et al [39] showed that the patients with atrial fibrillation have a greater increase in atrial size than those without atrial fibrillation. Recently, it has also been shown that elite junior athletes and collegiate football athletes have a significantly greater left atrial diameter [9,40]. Left atrial dimension (cm/m2 body surface area) was not significantly different in the elite basketball players in this study.

In this study, we found that the body height, body weight and body surface area were increased in the elite basketball athletes as compared with healthy sedentary subjects. Moreover, the body height, body weight and body surface area were positively associated with P wave dispersion. This relation may be due to increasing heart weight and size in athletes than in controls as in our study [41,42]. The duration of the P wave reflects the activation of atrial muscle and may depend primarily upon the mass of tissue excited. Thus, the P wave dispersion should be correlated with body height and body weight.

Increase of the body weight is an important factor of cardiovascular diseases. It is related to ventricular tachyarrhythmias and atrial fibrillation [43]. And it is also associated with metabolic syndrome including type 2 diabetes mellitus, hypertension, hyperlipidemia, insulin resistance and endothelial dysfunction [44]. Increased body weight is one of the most important determinants of left atrial size [45]. Left atrial enlargement, which is a powerful precursor of atrial fibrillation, may contribute to the increase in P wave duration and dispersion associated with increased body weight. It has been reported previously that changes in left atrial dimension and pressure may influence P wave duration [22]. In obese patients, left atrial enlargement and electrical instability may be caused by elevated plasma volume, ventricular diastolic dysfunction, insulin resistance and enhanced neurohormonal activity [46]. In recently, Duru M et al showed that [46] substantial body weight loss (at least a 10% loss of their original weight) in obese subjects is associated with a decrease of P wave dispersion and improvement in atrial repolarization abnormalities. Decreased sympathetic activity, decrease of hyperinsulinemia, and improvement in relative subendocardial ischemia after weight loss in obese subjects may contribute to the decrease in the level of P wave duration and dispersion [46,47].

In conclusion, P wave dispersion was increased in elite woman basketball players as compared with healthy sedentary subjects. P wave dispersion was correlated with heart rate, body height, body weight and body surface area.

## Study limitations

There might be some limitations in this study. Firstly, we did not measure sympathetic and parasympathetic system activation. Secondly, P wave dispersion measurement errors done with manual evaluation may be potential bias for observed results. However, manual measurement of P wave dispersion has been well accepted and used in several studies [19,20].

## Figures and Tables

**Table 1:**
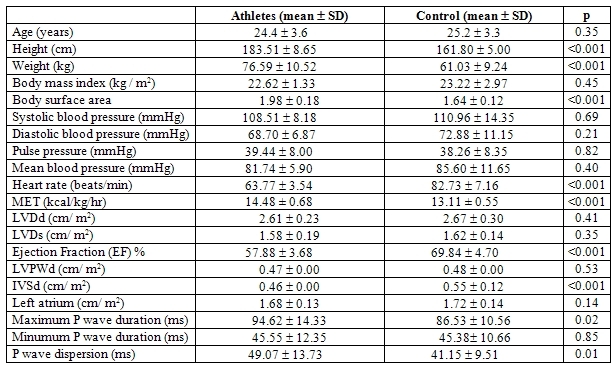
Anthropometric,hemodynamic and electrocardiographic values in groups
